# Genomic diversity and comprehensive taxonomical classification of 61 *Bacillus subtilis* group member infecting bacteriophages, and the identification of ortholog taxonomic signature genes

**DOI:** 10.1186/s12864-022-09055-w

**Published:** 2022-12-16

**Authors:** Haftom Baraki Abraha, Jae-Won Lee, Gayeong Kim, Mokhammad Khoiron Ferdiansyah, Rathnayaka Mudiyanselage Ramesha, Kwang-Pyo Kim

**Affiliations:** 1 Department of Food Science and Technology, Jeonbuk National University, Jeonju, 54896 South Korea; 2 Department of Agricultural Convergence Technology, Jeonbuk National University, Jeonju, 54896 South Korea

**Keywords:** Bacteriophages, Taxonomic classification, Intergenomic similarities, Signature genes, Orthologs, *Bacillus* phages, *B. subtilis* group, Phylogenetics

## Abstract

**Background:**

Despite the applications of *Bacillus subtilis* group species in various sectors, limited information is available regarding their phages. Here, 61 *B. subtilis* group species-infecting phages (BSPs) were studied for their taxonomic classification considering the genome-size, genomic diversity, and the host, followed by the identification of orthologs taxonomic signature genes.

**Results:**

BSPs have widely ranging genome sizes that can be bunched into groups to demonstrate correlations to family and subfamily classifications. Comparative analysis re-confirmed the existing, BSPs-containing 14 genera and 21 species and displayed inter-genera similarities within existing subfamilies. Importantly, it also revealed the need for the creation of new taxonomic classifications, including 28 species, nine genera, and two subfamilies (*New subfamily1* and *New subfamily2*) to accommodate inter-genera relatedness. Following pangenome analysis, no ortholog shared by all BSPs was identified, while orthologs, namely, the tail fibers/spike proteins and poly-gamma-glutamate hydrolase, that are shared by more than two-thirds of the BSPs were identified. More importantly, major capsid protein (MCP) type I, MCP type II, MCP type III and peptidoglycan binding proteins that are distinctive orthologs for *Herelleviridae*, *Salasmaviridae*, *New subfamily1*, and *New subfamily2*, respectively, were identified and analyzed which could serve as signatures to distinguish BSP members of the respective taxon.

**Conclusions:**

In this study, we show the genomic diversity and propose a comprehensive classification of 61 BSPs, including the proposition for the creation of two new subfamilies, followed by the identification of orthologs taxonomic signature genes, potentially contributing to phage taxonomy.

**Supplementary Information:**

The online version contains supplementary material available at 10.1186/s12864-022-09055-w.

## Background

Bacteriophages (phages) represent the most abundant biological entities in the biosphere. They are bacteria-infecting viruses, mostly containing dsDNA packaged into capsid/head. The genome size of tailed phages varies broadly in size, ranging from 10 kb to over 500 kb [1].

Virus taxonomy deals with the classification and nomenclatures of viruses into taxa to achieve better organization, stability, and predictability [2]. Phage classification supports an efficient way of genome data organizations in public databases with effective searching methods [3]. Ideally it shows evolutionary history among phages and facilitates comparative studies and the identification of new phages [4]. It also helps to understand the diversity and relationship of phages as well as to avoid confusion.

The current genome-based phage classification system [5] is still challenged by the ever-increasing number of phage genomes deposited by the scientific community, resulting in the accumulation of ICTV (The International Committee on Taxonomy of Viruses)-unclassified phages at different ranks in the NCBI databases [6]. For instance, only 21 out of 61 publicly available *Bacillus subtilis* group member species-infecting phages (BSPs) are ICTV-accepted species, which limits the classification study of newly discovered phages and may create unnecessary confusion.

Comparative genomics of large collections of phages is prominently reported using phages of pathogenic hosts, such as, 79 *Gordonia* phages [7], 223 *Pseudomonas* phages [8], 60 *Erwinia/Pantoea* phages [9], and 30 *B. cereus* group phages [10]. Moreover, there are several in silico studies using large collections of pathogen prophages [11–14].

When it comes to the industrially important, non-pathogenic species, in silico analysis using prophages of *Lactobacillus* and *B. subtilis* are reported, for instance, 27 intact prophages from 19 *Lactobacillus brevis* strains [15], 1459 intact prophages from 16 different *Lactobacillus* species [16], and 172 intact prophages from 164 *B. subtilis* [17]. However, studies using large numbers of actually-isolated and sequenced BSPs remains scarce.

Given the influence of phages on their hosts coupled with the importance of *B. subtilis* group species in food industries and other sectors, and being a member of the genus *Bacillus* along with the medically important human pathogen *B. anthracis* and *B. cereus* [18], studies on BSPs are welcoming.

Currently, there are a substantial number of BSPs whose complete genomes have been sequenced and can be studied exclusively. In this study, using various computational tools we carried out genome- and proteome-based studies and phylogenetic analysis on 61 BSPs. Furthermore, pangenome analysis was performed to identify the most shared orthologs and taxon-distinctive signature genes.

## Results

### General information of BSPs

A total of 61 BSPs’ genomes were collected from NCBI taxonomic database along with their taxonomic classification information. These phages are distributed into five families with some of them having subfamilies (Table 1). Also there are 14 genera that contain BSPs. Twenty-one of the 61 BSPs have ICTV-accepted species name, whereas the remaining 40 does not (Additional file 1).

**Table 1:** Sixty-one BSPs sorted according to their genome sizes

Genome-size	BSP	Accession No.	Length (kb)	Proteins	GC %	Subfamily	Family/Class	Genome-size	BSP	Accession No.	Length (kb)	Proteins	GC %	Subfamily	Family
Small-size	Goe1	NC_049975	18.38	24	37.49	*Picovirinae*	*Salasmaviridae*	Large-size	SP8	MW001214.1	138.74	209	40.30	*Spounavirinae*	*Herelleviridae*
	B103	NC_004165	18.63	17	37.66				SP10	NC_019487	143.99	236	40.49		
	Nf	NC_049976	18.75	27	37.32				Goe2	KY368639.1	146.14	226	40.26		
	Goe6	NC_049965	19.11	26	39.78				CampHawk	NC_022761	146.19	231	40.20		
	BSTP4	MW354668.1	19.15	31	39.95				SIOphi	NC_042133	146.7	206	39.02	*Bastillevirinae*	
	phi29	NC_011048	19.28	27	39.99				phi18	MZ751040.1	147.3	226	40.56	*Spounavirinae*	
	PZA	NC_001423	19.37	27	39.66				Goe10	MT601271.1	149.54	223	40.35		
	BSTP6	MW354670.1	19.37	32	39.78				Goe9	MT601270.1	150.96	225	40.36		
	GA1	NC_002649	21.13	35	34.65	*Tatarstanvirinae*			BSP38	NC_048726	153.27	254	41.75	*Bastillevirinae*	
	Gxv1	NC_049974	21.78	34	39.70	*Picovirinae*			BSTP3	MW354667	153.75	276	42.10		
	Stitch	NC_031032	24.32	36	30.36	*Northropvirinae*			BSP10	MF422185.1	153.77	236	42.10		
Medium-size	phi105	NC_004167	39.33	51	42.69		*Siphoviridae*		015DV002	MW419086.1	153.88	256	38.71		
	Ray17	MH752385.1	43.73	74	44.55				000TH009	MW419085.1	155.19	266	38.64		
	SPP1	NC_004166	44.01	97	43.72				000TH008	MW419084.1	155.25	266	38.61		
	049ML003	MN176228.1	44.82	81	43.67				phiNIT1	NC_021856.1	155.63	219	42.12		
	019DV004	MN176221.1	45.24	84	42.64				BSP9	MG000860.1	155.96	228	42.01		
	049ML001	MN176227.1	45.24	82	43.75				Goe3	NC_048652	156.43	246	41.93		
	000TH010	MN176219.1	46.28	90	43.84				015DV004	MW419087.1	156.58	270	38.58		
	PM1	NC_020883	50.86	86	41.29		*Myoviridae*		Grass	NC_022771	156.65	252	42.25		
	274BB002	MZ501264.1	52.37	81	42.51		*Siphoviridae*		278BB001	MZ501265.1	158.56	269	42.05		
	276BB001	MN176231.1	52.37	81	42.51				Goe7	MN043730.1	158.67	251	41.84		
	056SW001B	MN176230.1	52.69	83	42.55				043JT007	MZ501263.1	161.64	284	42.01		
	019DV002	MN176220.1	52.75	84	42.64				010DV005	MZ501261.1	161.88	282	41.98		
	268TH004	MW394467.1	52.83	82	42.45				010DV004	MZ501260.1	163.32	281	41.98		
	BSTP12	MW354678.1	55.42	80	39.91		*Podoviridae*		035JT004	MZ501262.1	163.32	286	41.88		
	BSP7	MH707430.1	55.46	70	39.92			Extra-large size	SP15	NC_031245	221.91	317	38.61		*Myoviridae*
Large-size	Goe12	MT601273.1	124.29	174	34.80		*Caudoviricetes (Siphoviridae)*		AR9	NC_031039	251.04	291	27.75		
	BUCT082	MZ969646.1	125.02	180	34.79				PBS1	NC_043027	252.20	311	27.71		
	Goe11	MT601272.1	126.29	175	34.91				Minimum value		18.38	17	27.71		
	Goe13	MT601274	126.85	179	34.79				Maximum value		252.20	317	44.55		
	phi3T	KY030782	128.38	201	34.96				Average		105.23	163	39.67		
	SPO1	NC_011421	132.56	204	39.96	*Spounavirinae*	*Herelleviridae*		Median		128.382	182.50	40.30		
	SPbeta	NC_001884	134.42	185	34.64		*Caudoviricete (Siphoviridae)*								

The genome sizes of the BSPs range from as low as 18.38 kb (B103) to as high as 252.20 kb (PBS1) with an average size of 105.23 kb (128.38 median). Based on genome size, the BSPs can be bunched into four groups, i.e., those with small-size genomes (18.38–24.32 kb, ave. 19.93 kb), medium-size (39.33–55.46 kb, ave. 48.89 kb), large-size (124.29–163.32 kb, ave., 148.16 kb) and extra-large-size (221.91–252.20 kb, ave., 241.72 kb) genomes (Table 1).

**Fig. 1:**
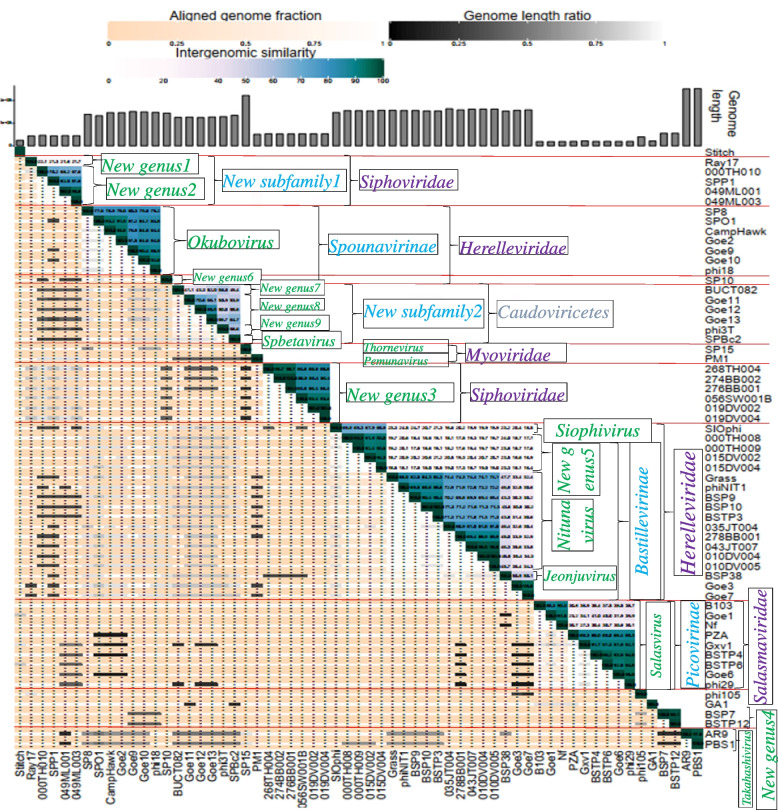
Intergenomic similarity analysis of 61 BSP genomes. The right half of the heatmap represents intergenomic similarities. The background color of the clusters which is green, denotes the degree of similarity, with the dark green indicating identical or highly similar genomes. The left hand represents indicator values for the aligned fraction of each genome pair and genome length ratio. The darker colors emphasize low values, indicating either a small fraction of the genome was aligned (orange to white color gradient), or there is a high difference in the length of the two genomes (black to white color gradient). The top side indicates genome length and annotations

All BSPs in the family *Salasmaviridae* have small-size genomes, while those of the *Herelleviridae* have large-size genomes. BSP members of the *Siphoviridae* and *Caudoviricetes* have medium and large-size genomes, respectively. The BSP members of the *Myoviridae* have extra-large-size genomes, except for the phage PM1, which has medium-size genome. BSPs organized under the family *Podoviridae* were categorized into the medium-size group (Table 1, Fig. 1).

The protein numbers of the BSPs are found in the range of 17 (B103) to 317 (SP15) with an average of 162 proteins (182.50 median). The GC-contents of the BSPs span from 27.71% (PBS1) to 43.84% (000TH010) with an average value of 39.63% (40.28 median) (Table 1).

The classification analysis of the BSPs was performed by gathering BSPs from the NCBI followed by comparison with their current classification status in the current ICTV virus taxonomy release (2021 release), if existing, or in the 2020 release, if not existing. Worthy of mentioning, morphology-based family-level classifications (*Siphoviridae*, *Myoviridae* and *Podoviride*) are abolished in the latest 2021 taxonomy release, unlike in the 2020 release. A significant fraction of BSPs do not appear in the 2021 taxonomy release as they became family-level-unclassified.

### Genome-based classification of the BSPs

#### Intergenomic similarity analysis

Intergenomic similarities among the genomes of the 61 BSPs were calculated using the VIRIDIC tool, which computes based on BLASTN algorithms. The result was then evaluated in accordance with the current ICTV phage classification scheme to confirm existing classifications and propose new classifications for BSPs that are not ICTV-accepted (Fig. 1 and Additional file 2).

##### Species-level classification and the creation of 28 new species

Twenty-one of the 61 BSPs are classified into species-level having an ICTV-accepted species names (Additional file 1). Intergenomic similarity analysis confirmed the classification of 19 of these existing species where they showed greater than 5% intergenomic distances. Worthy of note, two existing species, namely, the phi29 and Goe6, showed a boundary similarity of 95.0% (Fig. 1, Additional file 2).

Furthermore, the analysis revealed 28 new species fulfilling the species cutoff value, i.e., ‘more than 95%’ in overall nucleotide sequence similarity as established by the ICTV. Some of the newly proposed species contain only one member and others more than two BSPs (Fig. 1). Thirty-eight BSPs were classified into the new species, while two, the AR9 and Goe7 were classified into existing species of PBS1 and Goe3, respectively.

##### Genus-level classification and creation of nine new genera

Currently, there exist 14 ICTV-accepted genera into which BSPs are distributed (Additional file 1). The current intergenomic similarity analysis re-confirmed these existing genera and further showed the need for the creation of nine new genera, which are designated as *New genus1* through *New genus9* in this study. The creation of the new genera is justified in line with the nucleotide sequence identity cutoff set for genera by the ICTV, i.e., 70%.

##### Subfamily classification and proposition to create two new subfamilies

There are five existing subfamilies to which BSPs are assigned. These are *Bastillevirinae* and *Spounavirinae* of the *Herelleviridae*, and *Northropvirinae*, *Picovirinae*, and *Tatarstanvirinae* of the *Salasmaviridae* (Table 1). The current intergenomic analysis showed inter-genera similarities in the range of 16.4–50.8% for the *Bastillevirinae* and 23.2–41.0% between two genera of the *Picovirinae* (Fig. 1, Additional file 2). No inter-genera similarity was observed for the *Spounavirinae*, while the *Northropvirinae* and *Tatarstanvirinae* contain single BSP-containing genera.

Further analysis showed inter-genera similarities of 21.3–22.1% between *New genus1* and *New genus2* under the *Siphoviridae*, which led to the proposition of the creation of a new subfamily designated as “*New subfamily1*”. Also, 41.1–64.7% similarities among four genera (*Spbetavirus*, *New genus7* through *New genus9*) led to the proposition of the creation of a 2nd new subfamily designated as “*New subfamily2*” in this study (Fig. 1).

Beyond the intergenomic sequence similarity, more detailed characteristics of the BSP members of the newly proposed subfamilies are shown in Table 2, which could be considered as an additional evidence in support to the establishment of the new subfamilies. The *New subfamily1* formed a monophyletic group in the proteomic-based phylogeny analysis as well as in phylogenetic analysis using the MCP (See below, Figs. 4, 5, and 7). On the other hand, in the phylogenetic analysis using tail fiber proteins (the topmost BSP-shared ortholog) one member (Ray17) was separated from the group, instead PM1 was joined to the group (data not shown). Similarly, the *New subfamily2* was formed a monophyletic group during the phylogenomic, proteome-based phylogeny and phylogenetic analysis using the peptidoglycan binding protein but not using DNA polymerase.

**Table 2:** Characteristics of BSP members of the newly proposed *New subfamily1* and *New subfamily2*

Family/Class		*Siphoviridae*		*Caudoviricetes*			
Proposed subfamily		*New subfamily1*		*New subfamily2*			
Member Genus		*New genus1*	*New genus2*	*Spbetavirus*	*New genus7*	*New genus8*	*New genus9*
Member Species		1. Ray17	2. 000TH010	1. SPbeta	2. BUCT082	3. Goe11	6. phi3T
			3. SPP1			4. Goe12	
			4.049ML001/049ML003			5. Goe13	
Inter-genera similarity %		21.3–22.1		41.1–64.7			
Phylogenomy		Non-monophyletic		Monophyletic			
Proteome-based cluster/phylogeny		Distinct cluster/Monophyletic		Distinct cluster/Monophyletic			
Subfamily-unique genes/proteins using 30% identity and 50% coverage cutoff		Major capsid, HTH DNA binding domain and portal protein, and recombinase, transcription factor, terminase large subunit, and transcriptional repressor		Integrase, tyrosine recombinase, lysogeny pheromone peptide, arbitrium peptide, UV damage repair protein, ssDNA specific exonuclease, SOS response-associated peptidase, Lysis-lysogeny pheromone receptor			
Phylogenetic analysis	MCP	Monophyletic		MCP is absent in all members			
	DNA polymerase	DNA polymerase is absent in all members		Non-monophyletic			
	Tail fiber protein	Not monophyletic		Monophyletic except phi3T which lacks the protein			
	PGA hydrolase	Monophyletic except SPP1 that lacks the protein		Monophyletic except phi3T which lacks the protein			
	Peptidoglycan binding protein	The protein is absent in all members		Monophyletic			
	Genome-size group	Medium (39.33–55.46 kb)		Large (124.29–163.32 kb)			

#### Phylogenomic and synteny analysis

**Fig. 2:**
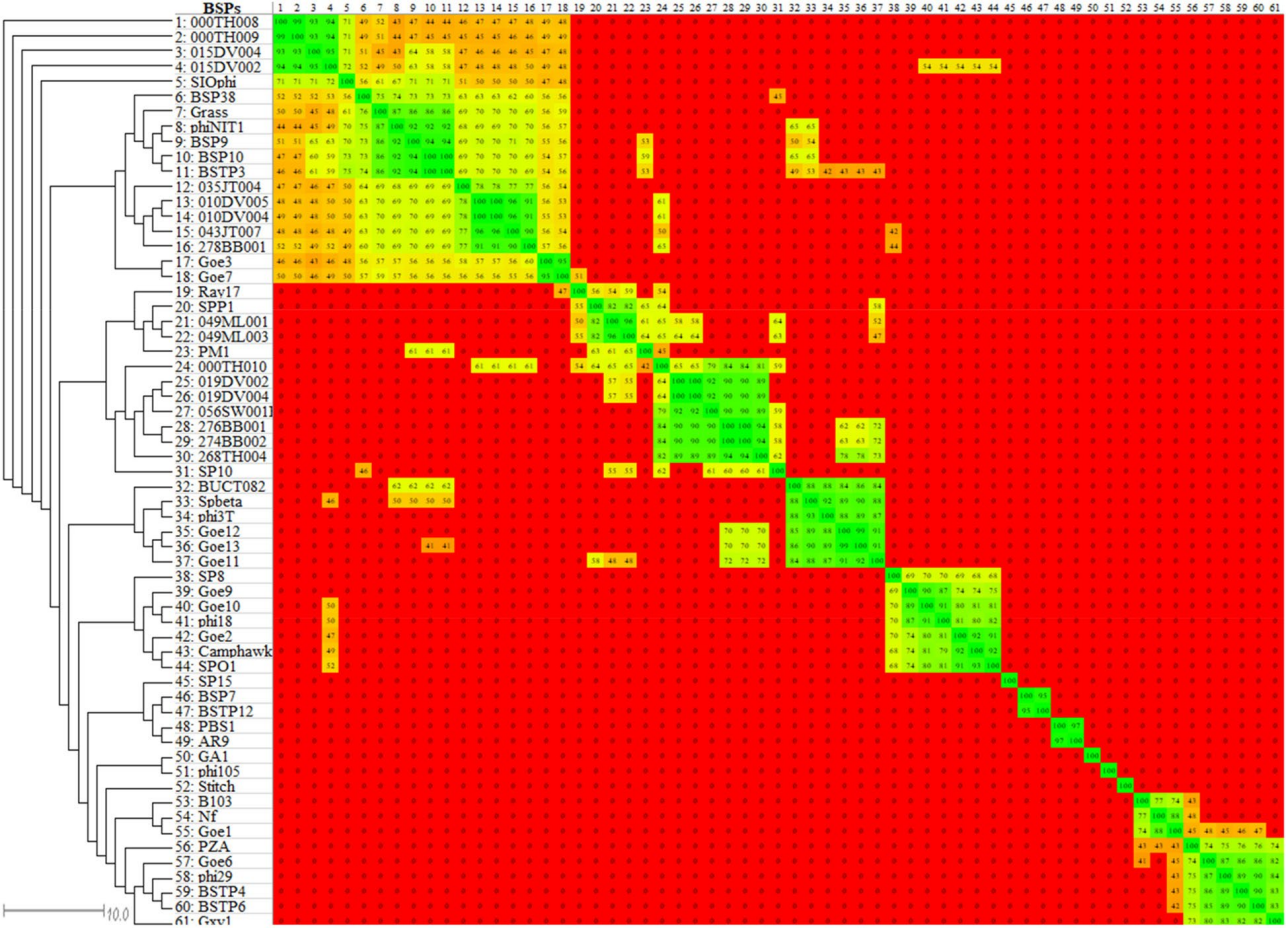
Phylogenomic relationships of 61 BSPs. The green, yellow, and red colors depict high, medium, and low/no similarities, respectively. The genus, subfamily, and family of each phage as retrieved from the NCBI taxonomic database is indicated on the right side

Phylogenomic analysis of the BSPs showed neither family-level distinct clusters nor monophyletic groups. In general, separate clusters were formed at subfamily-level for the *Bastillevirinae* and *Picovirinae*. BSPs belonging to the family *Siphoviridae* formed three clusters. The phage PM1 was clustered together with the members of the family *Siphoviridae* in the phylogenomic analysis (Fig. 2 and Additional file 3).

**Fig. 3:**
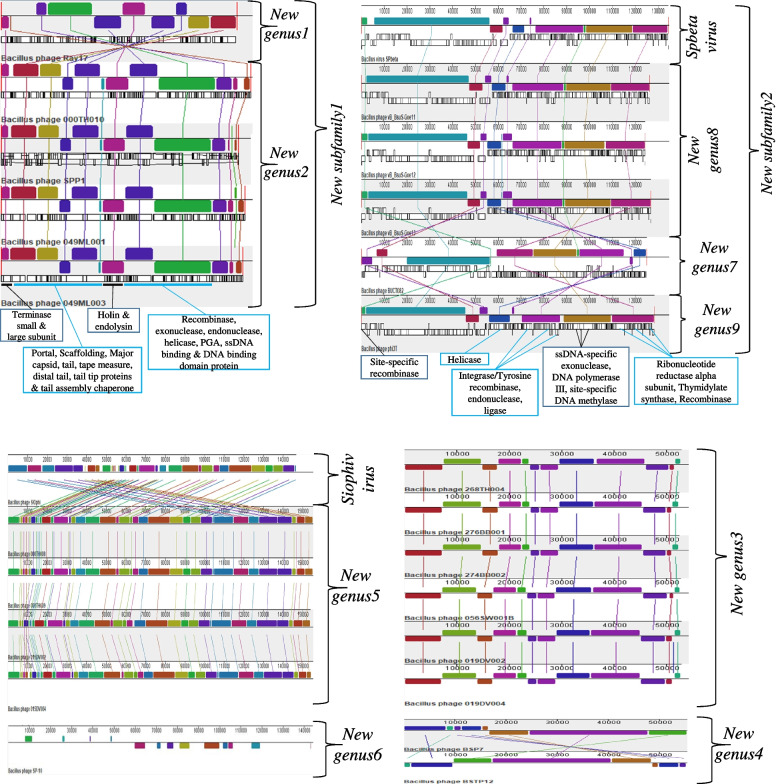
Genomic synteny analysis of 61 BSPs. Only the synteny analysis of BSPs that are assigned to the newly created genera are presented. In the genomic blocks some genes’ locations for the *New subfamily1* and *New subfamily2* are indicated

Genomic synteny analysis was carried out to illustrate gene organizations of the BSP genomes and to align them with their classifications. Overall, conserved gene organizations were observed for BSP members of a genus supporting their classifications together. The *New genus2*, *New genus3, New genus5* and *New genus8* showed conserved gene organization, while the gene organization in the *New genus4* was not conserved (Fig. 3).

### Proteome-based classification of the BSPs

According to the proteome-based clustering using tBLASTx program, no significant similarity was observed at family-level. The existing subfamilies *Bastillevirinae* and *Spounavirinae* of the *Herelleviridae* were clustered separately with no significant inter-subfamily similarity. On the other hand, as high as 22% inter-subfamily similarity was observed for the *Salasmaviridae*.

The intra-subfamily similarity was greater than 35% for the *Bastillevirinae* but insignificant (< 14%) for the *Spounavirinae*. A minimum of 48% intra-subfamily similarity was observed for the *Picovirinae*.

**Fig. 4:**
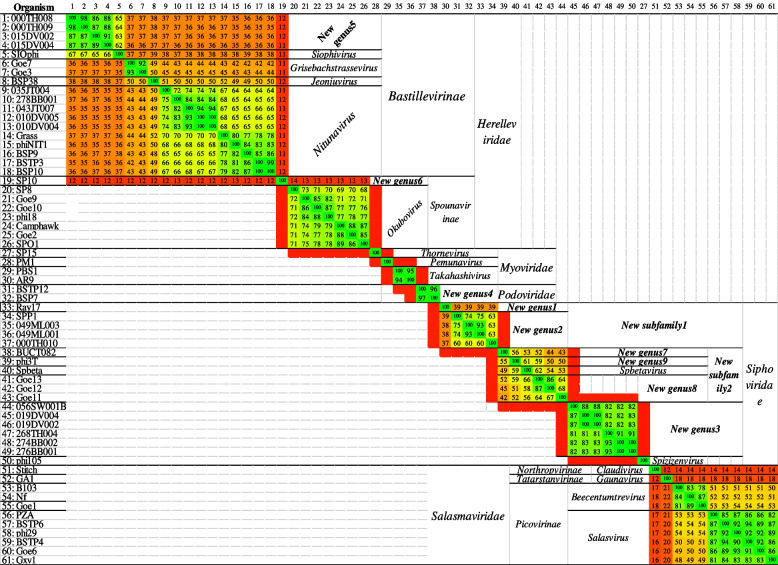
Proteome-based clustering of 61 BSPs. The green, yellow, and red colors represent similarity degree from identical/high similarity to low/no similarity

The analysis also revealed significant intra-subfamily similarities among the newly proposed subfamilies. The *New subfamily1* had more than 37% intra-subfamily similarity, while *New subfamily2* had more than 42%. Moreover, they formed a clearly-defined cluster, supporting their establishments (Fig. 4 and Additional file 4).

**Fig. 5:**
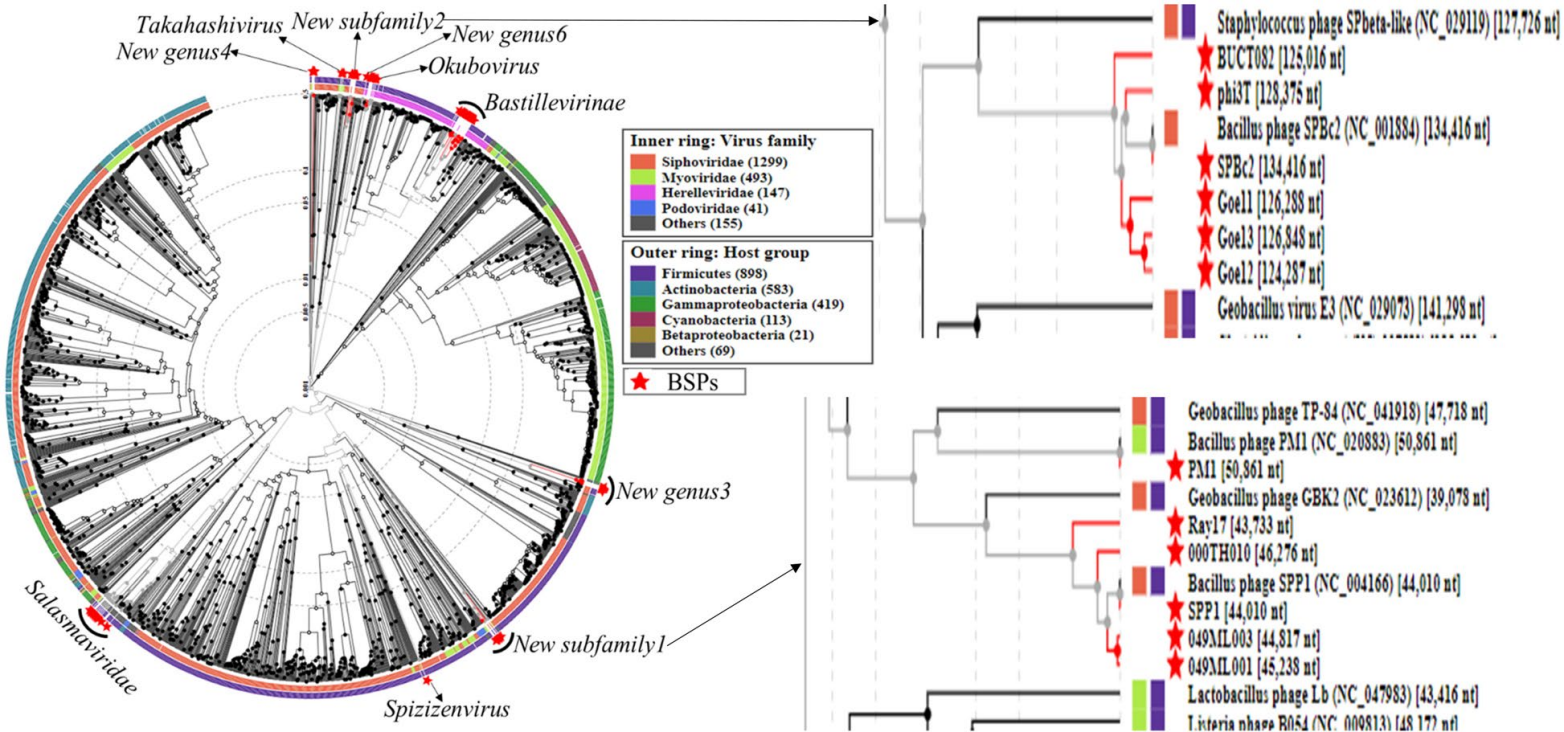
Proteomic tree generated using prokaryotic dsDNA viruses as references. The red stars represent the 61 BSPs. The inner and outer colored rings represent morphotypes and host groups, respectively. The *New subfamily1*, *New subfamily2* and phylogenetically closest neighbors are zoomed in to the right side

Furthermore, the proteomic tree of the 61 BSPs was generated by the ViPTree server making use of the prokaryotic dsDNA reference viruses provided by the server. Accordingly, BSPs belonging to different families and subfamilies were clustered separately, similar to that of the genome- and proteome-based clustering results. The clusters of all the BSPs were built in the range of phages that infect the phylogenetically proximal host group in the phylum Firmicutes (Fig. 5).

Members of the *New subfamily1* and *New subfamily2* were found to be monophyletic. The *Geobacillus* phage GBK2 (NC_023612) [39.09 kb] was phylogenetically closest neighbor to the *New subfamily1* followed by phage PM1. Likewise, *Staphylococcus* phage SPbeta-like (NC_029119) [127.73 kb] was phylogenetically closest neighbor to the *New subfamily2* (Fig. 5).

### Pangenome analysis of the BSPs

**Fig. 6:**
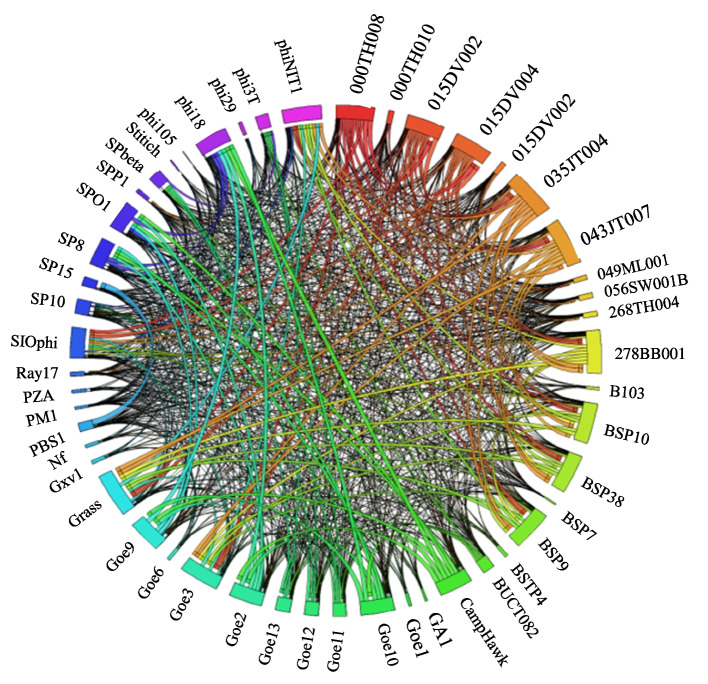
The map of the pangenome analysis of the BSP species as depicted using circus. The colored ribbons depict pairwise protein similarity of the BSPs based on the bit score of blast alignment. The circular segment depicts the row-column values. The segments are labelled with the row names of the BSPs. The size of the angular segment shows the proportion of the total interactions

Sequence similarity and coverage cutoff of 30 and 50%, respectively, were considered for pangenome analysis of the BSPs as recommended [5]. The protein sequences of most BSP species showed intense similarity interactions with each other. Whereas some BSP species, primarily those belonging to the *Salasmaviridae* and *New subfamily1* demonstrated fewer similarity interactions (Fig. 6).

The analysis revealed inter-family level shared orthologs. BSP genes encoding the tail fiber/spike proteins were the topmost inter-family orthologs shared by 75.41% (46/61) of the BSPs including from all families except *Salasmaviridae*. Likewise, poly-gamma-glutamate (PGA) hydrolase encoding genes were the 2nd orthologs shared by 72.13% (44/61) of the BSPs belonging to different family-level clusters (Additional file 5).

Looking into more details, the pangenome analysis showed that all members of the family *Herelleviridae* (*n* = 26) shared a significant number of orthologs. These include genes encoding the major capsid protein (MCP), DNA polymerase, nuclease, junction resolvase, RNA polymerase sigma factor, tail sheath protein, tail protein, and baseplate assembly.

Similarly, all BSP members of the *Salasmaviridae* (*n* = 11) have shared orthologs, including genes for dsDNA binding protein, lower collar protein, tail protein, major head protein, DNA polymerase, upper collar protein, and DNA encapsidation protein. In contrast, no orthologs shared by all BSP members of the *Siphoviridae* (*n* = 18, including the siphovirus SPbeta and its related BSPs in the *Caudoviricetes*) were identified (Additional file 5).

The MCP, transcription factor, transcriptional repressor, terminase large subunit were shared orthologs for the *New subfamily1* while integrase, tyrosine recombinase, lysogeny pheromone peptide, UV damage repair protein, ssDNA specific exonuclease, and Lysis-lysogeny pheromone receptor were shared orthologs for *New subfamily2* (Additional file 5).

Some of the BSPs appeared to have co-orthologs, two or more genes that result from lineage-specific duplications followed by speciation [19]. Most of the BSPs with co-orthologs were from the family *Herelleviridae*, including 000TH008, 000TH009, 010DV004, 010DV005, 035JT004, 015DV002, 015DV004, 043JT007, 278BB001, CampHawk, Goe2, Goe7, Goe9, Goe10, BSP38, and phi18. In addition, six BSPs from the *Siphoviridae*, namely, 019DV002, 019DV004, 056SW001B, 268TH004, 274BB002, and 276BB001, as well as one from the *Salasmaviridae* (Gxv1) have co-orthologs. Furthermore, most (48/61) of the BSPs have genes without orthologs.

### Phylogenetic analysis of the BSPs

Following pangenome analysis of the BSPs, phylogenetic analysis was continued using MCP and DNA polymerase. The dataset was prepared by searching the gene of interest from the annotations of the BSPs, through blasting using genes of related phages. In the cases where the gene of interest is not found, both structural and functional re-annotation was performed, followed by searching for the gene of the interest. Nevertheless, MCP could not be found for eight BSPs, including six temperate BSPs of the *New subfamily2* (SPbeta, phi3T, Goe11, Goe12, Goe13, and BUCT082) as well as BSP7 and BSTP12. Likewise, we could not find DNA polymerase for five BSP members of the *New subfamily1* (SPP1, Ray17, 000TH010, 049ML001, 049ML003), phi105, and PM1.

**Fig. 7:**
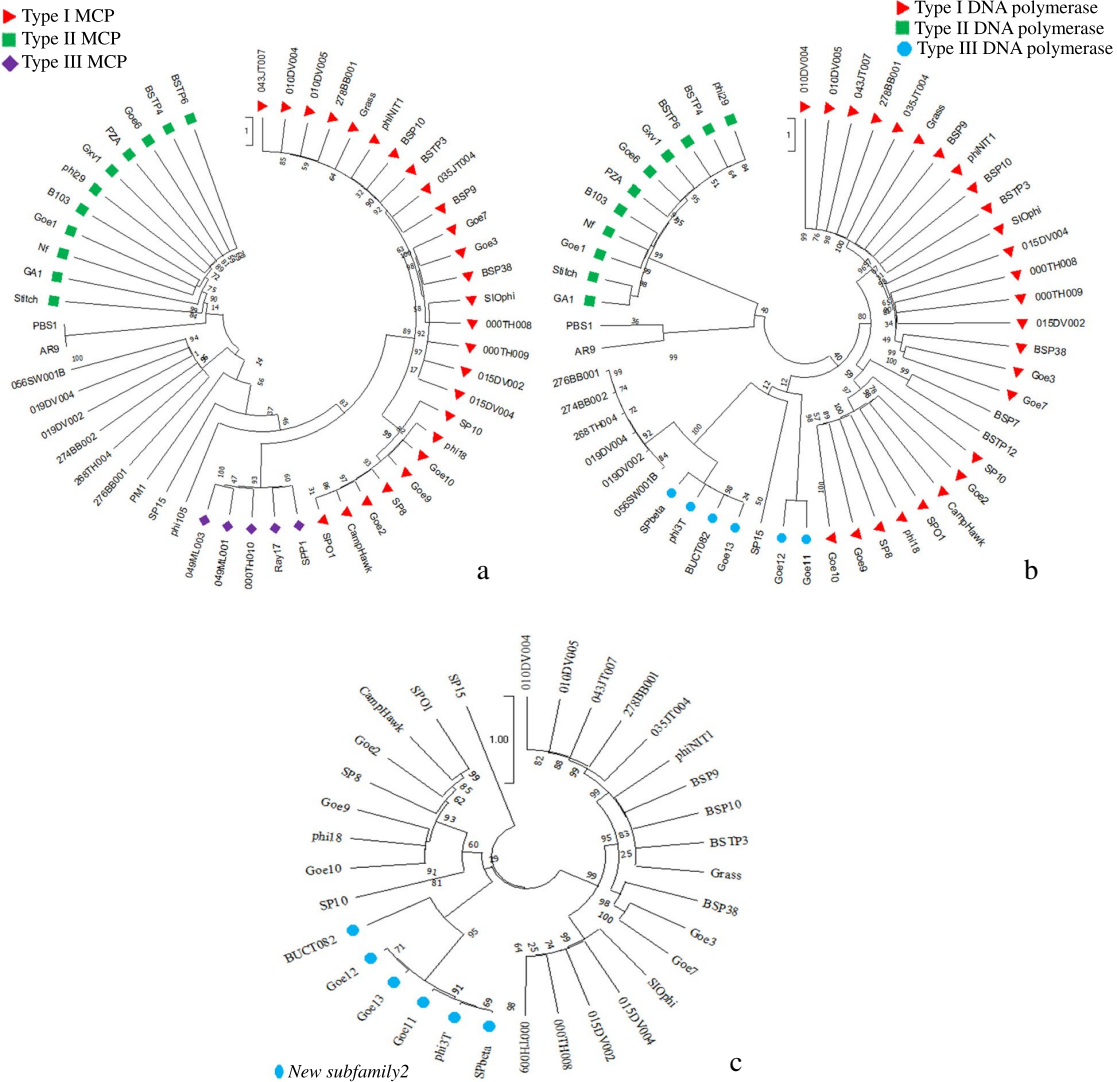
Evolutionary history analysis of the 61 BSPs conducted in MEGA X using sequences of MCP (**a**), DNA polymerase (**b**) and peptidoglycan binding protein (**c**). The red triangles represent BSP members of the *Herelleviridae* while the green rectangles represent BSP members of the *Salasmaviridae*. The purple diamonds illustrate BSP members of the New subfamily1. The blue circles represent members of the *New subfamily2*. The MCP and DNA polymerase are also typed based on their evolutionary relationships

According to the phylogenetic analysis, all BSPs of the *Herelleviridae* were found a monophyletic using MCP that is labelled as MCP Type I (Fig. 7a). All BSPs of the *Salasmaviridae* formed a monophyletic cluster using MCP that is labelled as MCP Type II (Fig. 7a), and DNA polymerase labelled as DNA polymerase Type II (Fig. 7b).

Moreover, the BSP members of the *New subfamily1* formed a monophyletic cluster using MCP (labelled as MCP Type III) (Fig. 7a). The BSP members of the *New subfamily2* did not formed a cohesive monophyletic group using the DNA polymerase that is labelled as DNA polymerase Type III (Fig. 7b).

Phylogenetic analysis was carried out using orthologs sequences that are shared by BSPs in different family-level clusters, namely, peptidoglycan binding, tail fiber/spike, and PGA hydrolase proteins. In the analysis using peptidoglycan binding protein, *New subfamily2* formed a monophyletic cluster using peptidoglycan binding protein (Fig. 7c).

Generally, the BSP members of different subfamilies clustered separately when the tail fiber/spike and PGA hydrolase proteins were used for phylogenetic analysis. Using fiber/spike protein, the Ray17, a member of the *New subfamily1* was replaced by PM1 (a non-member) and formed a monophyletic group (data not shown).

## Discussion

In the current study, we used 61 BSPs (of which only 21 are ICTV-accepted species) organized under five families (the *Herelleviridae*, *Siphoviridae*, *Salasmaviridae*, *Myoviridae*, and *Podoviridae*) in the NCBI taxonomic databases. A number of genome- and proteome-based computational approaches were taken to show the genomic diversity and taxonomical classifications of the BSPs. Furthermore, their entire set of genes were studied for possible identification of ortholog genes (orthologs) that are shared by all or most of the BSPs and taxon-based signature genes.

BSPs have a widely ranging genome size (18.38–252.20 kb) which can be bunched into small (18.38–24.32 kb), medium (39.33–55.46 kb), large (124.29–163.32 kb), and extra-large (221.91–252.20 kb) genome-size groups. All *Salasmaviridae* (*n* = 11) members were classified as small, while *Herelleviridae* (*n* = 26) members were classified as large, demonstrating a clear correlation between genome-size and classification. The genome sizes of *Salasmaviridae* and *Herelleviridae* were reported to be between 18 and 27 kb [20] and 125–170 kb [21], respectively. Members of the *Myoviridae* fitted into the extra-large size except phage PM1, joining the medium-size group along with the members of the *Siphoviridae*. The PM1 was originally reported to be in the *Siphoviridae* [22], which needs further clarifications. *Siphoviridae* members are categorized as medium-sized. The siphovirus SPbeta and its related BSPs of the *New subfamily2*, however, appeared to have a large-sized genome. A cohesive genome size-taxon correlation may be helpful to speculate the likely classification of newly discovered BSPs based its genome size.

Genome-based phage classification enables easier management of public databases and identification of new phages [23] and eventually facilitates further phage-based studies and applications. In response to the influence of genome sequencing on phage taxonomy, the ICTV has introduced a genome-based taxonomical classification where 95 and 70% genome-level nucleotide identities are used as species-and genus-demarcation criteria, respectively. Furthermore, subfamilies are to be created when distinct genera are related below the family level [5].

The BSPs’ intergenomic similarity analysis revealed high genomic diversity with no to identical similarity, resulting in sparsely populated lower-level taxonomic classifications. In line with the ICTV genome-based classification scheme, the analysis re-confirmed the existing 21 species and 14 genera. Importantly, it also showed the need for the creation of 28 new species and nine new genera, which add up to 49 species and 23 genera to accommodate all the BSPs. Seven of the existing and four of the new genera contained only a single member, suggesting the need for further isolation of BSPs to fully appreciate their diversity.

Currently, there are five BSP-containing ICTV-accepted subfamilies: the *Bastillevirinae* and *Spounavirinae* in *Herelleviridae*; *Northropvirinae*, *Picovirinae*, and *Tatarstanvirinae* in *Salasmaviridae*. The current intergenomic analysis showed an inter-genera similarity of 16.4–50.8% among five distinct BSP-containing genera of the *Bastillevirinae* and 23.2–41.0% among two distinct genera of the *Picovirinae* (Fig. 1). By the same token, in the *Siphoviridae* that have no existing subfamily, inter-genera similarities of 21.3–22.1% between two distinct genera and 41.1–66.1% among other four distinct genera were observed. This is further supported by observation from the proteome-based analysis where clusters under the *Siphoviridae* showed relatively less similarities to each other than those of the *Herelleviridae* and *Salasmaviridae* (Fig. 4). It is, therefore, worth considering the creation of subfamilies under the *Siphoviridae* to show their diversity. Consequently, the creation of *New subfamily1* and *New subfamily2* to accommodate the two and four inter-related genera, respectively, is proposed.

The *New subfamily1* contains the *New genus1* and *New genus2* (Ray17, SPP1, 000TH010, 049ML001, and 049ML003). All the members shared orthologs genes, including the MCP, recombinase, terminase large subunit, and transcriptional repressor. Remarkably, DNA polymerase is absent in all the members, which may be speculated that these phages can manipulate the host DNA polymerase [24].

The *New subfamily2* includes the genera *Spbetavirus* and *New genus7* through *New genus9* (SPbeta, phi3T, Goe11, Goe12, Goe13, and BUCT082). All these phages and phi105, which also belong to the *Siphoviridae*, are reported to be temperate phages [25–28]. The members of the *New subfamily2* share several orthologs genes, including integrase and tyrosine recombinase, whose functions can be related to lysogeny [29]. Unexpectedly, all members of the *New subfamily2* do not possess MCP, which is a structural protein essential to protect the fragile nucleic acid of the phages [30]. Kohm and Hertel [31] described that almost nothing is known about SPbeta virion assembly despite its age of more than 50 years, suggesting the need for further study. It’s important to note that the absence of DNA polymerase and MCP genes may not necessarily mean that phages do not have them. It could mean that there are no described homolog genes in the database.

While analyzing the BSPs, we observed inconsistencies from the perspective of the ICTV classification criteria and the various bioinformatics tools used. There is a case in which a member partially fulfilled the ICTV genus demarcation criteria (70% of genomic similarity) to be included in a genus. In the *New genus8* that contains Goe11, Goe12, and Goe13, the Goe11 showed greater than 70% similarity to Goe12 but less than 70% to Goe13, and the Goe12 showed greater than 70% similarity to Goe13 (Fig. 1). Looking into the genomic synteny analysis, Goe11 showed a slight difference from the other two members (Fig. 3). Whereas these members formed a distinct cluster during the proteome-based clustering and phylogeny (Fig. 4), they were not monophyletic during the phylogenetic analysis using DNA polymerase (Fig. 7b). In *New genus2* (000TH010, SPP1, 049ML001, and 049ML003), a similar pattern of inconsistency was observed, with 000TH010 showing greater than 70% similarity to SPP1, but less than 70% to the other members during the intergenomic analysis. Another inconsistency is that the phage PM1 formed a monophyletic group with the *New subfamily1* during the phylogenomic analysis (Fig. 2) but during the intergenomic similarity and proteome-based analyses, it was not a member.

Orthologs are genes originating from a single ancestral gene in the last common ancestor of the compared genomes and typically perform equivalent functions [32]. Because the BSPs’ hosts are phylogenetically closely related *B. subtilis* group species, one or more ortholog(s) that are shared-by-all can be expected. Such orthologs might be further analyzed for their possible use to mark BSPs from phages of other hosts. However, pangenome analysis using a 30% identity and 50% coverage cutoff did not reveal the presence of ortholog common to all the 61 BSPs.

Instead, the topmost shared orthologs that are common to more than two-thirds of the BSPs were identified. The tail fiber/spike encoding genes were the topmost shared ortholog, found in 75.41% of the BSPs of all families except the *Salasmaviridae*. The tail fibers/spike proteins facilitate initial recognition of a suitable host by interacting with bacterial surface receptors [33]. In other words, the BSPs carry similar types of tail fiber/spike proteins that may be used to infect different species within the *B. subtilis* group. Such BSPs may be less specific, thereby widening the range of their hosts—so do their effects on the hosts and fermentation processes.

The BSP gene encoding poly-gamma-glutamate (γ-PGA) hydrolase was the 2nd most shared ortholog, common to 72.13% of BSPs in different family-level clusters. The PGA hydrolase degrades γ-PGA. The γ-PGA is a *B. subtilis* fermented food component known for its various healthcare and industrial applications [34]. It is used for Calcium absorption, moisturizing, and as an immune-stimulating, anti-tumor, and super-absorbent polymer [35]. Phages use γ-PGA hydrolase to eliminate γ-PGA because it functions as a physical barrier to phage adsorption [36]. Indirectly, the BSPs can affect the quality of *B. subtilis* fermented foods undesirably by degrading the PGA, as demonstrated experimentally by Ghosh et al. [37]. With this in mind, the presence of γ-PGA hydrolase in most of the BSPs may suggest the importance of BSPs on the quality of *B. subtilis* fermented foods by affecting the beneficial traits of the hosts.

The discovery of taxon-distinctive phage genes (genetic signatures) is key to enabling the identification and taxonomic assignment of existing and new phages. We identified potential signature gene orthologs to family-level taxa that may serve as criteria for BSPs to include in or exclude from the family following phylogenetic analysis. The phylogenetic groups of such genes were characterized and include Type I MCP for the family *Herelleviridae* (26 members), Type II MCP and Type II DNA polymerase for *Salasmaviridae* (11 members). In all cases the members formed a monophyletic group in the phylogenetic analysis using the respective genes (Fig. 7).

Unlike the cases of *Herelleviridae* and *Salasmaviridae*, no orthologs specific to all BSP members of the *Siphoviridae* (*n* = 18) were identified. This indicates that the members are distantly related, necessitating the formation of new subfamilies, if possible, within the family for improved taxonomic classification and to show the diversity. With this in mind, we proposed two new subfamilies that contain two distinct clusters of genera under the family, followed by the identification and analyzing of possible signature genes. These subfamilies were designated as *New subfamily1* and *New subfamily2* in this study. Type 3 MCP and peptidoglycan binding proteins could serve as signatures for the *New subfamily1* and *New subfamily2*, respectively, in which each subfamily formed a monophyletic group using the respective genes.

## Conclusions

In conclusion, we studied 61 BSPs and showed high genomic diversity and widely ranging genome sizes; re-confirmed existing taxonomical classifications and proposed new classifications evaluated from genomic-diversity and genome-size perspectives; and identified shared orthologs and taxonomical signature genes.

## Methods

### Data collection

Sixty-one BSPs in the order of *Caudovirales* were collected from the taxonomic database maintained by the National Center for Biotechnology Information (NCBI)/GenBank (accessed on 14 October, 2021) (Table 1). They were then used for investigations of genetic diversity, taxonomic classification, and signature genes. The results of the BSPs’ classification analysis were compared to their current ICTV-classification status in order to confirm, if present, or suggest their creation, if not. The GC content of the genomes was calculated using GC-Profile software [38].

### Genome-based classification of BSPs

Intergenomic similarities among BSP genomes were computed using a Virus Intergenomic Distance Calculator (VIRIDIC) [39]. The reward and penalty scores for matching and mismatching bases, respectively, were set to 1 and − 2, i.e., the same as the default parameters of the NCBI_BLASTN. The species and genus threshold values were set to 95 and 70% intergenomic similarities, respectively.

Phylogenomic similarity matrix was generated using Gegenees software with fragment/step size of 200/100 and 40% threshold [40], followed by the exportation of a nexus file and used to generate phylogenomic tree by SplitsTree 4 using NJ method [4, 41]. The Synteny alignment and visualization was performed using progressiveMauve [42].

### Proteome-based clustering of BSPs

To get further supporting evidence for the genome-based classifications, proteome-based clustering tools were used, including ViPTree [43] and TBLASTX mode of the Gegenees software [40].

### Phylogenetic analysis of BSPs

Evolutionary history of the BSPs was inferred by using the Maximum Likelihood method and Dayhoff matrix based model with 1000 bootstrapping in MEGA X [44]. The analysis was done using major capsid protein (MCP), DNA polymerase, tail fiber/spike proteins, poly-gamma-glutamate (PGA) hydrolase, and peptidoglycan binding sequences.

### Pangenome analysis of BSPs

Pan-genomic features of the 61 BSPs were assessed by running Proteinortho V6 [19] on Linux-based Ubuntu operating system using less stringent criteria i.e., 30% sequence identity and 50% coverage as recommended by Turner et al. [5]. In the analysis, entire genes of the BSPs were analyzed including the orthologs, co-orthologs and genes without orthologs.

## Supplementary Information

**Additional file 1.** Existing and newly proposed classifications of 61 BSPs.

**Additional file 2.** Intergenomic similarities of 61 BSPs.

**Additional file 3.** Phylogenomic relationships of 61 BSPs.

**Additional file 4.** Proteome-based clustering of 61 BSPs.

**Additional file 5.** Pangenome analysis of 61 BSPs.

## Data Availability

The datasets used during the current study are available in figshare repository [10.6084/m9.figshare.21204890]. The data supporting the results of this article are included in the manuscript and its supplementary information files. The BSPs and accession numbers includes Goe1 [NC_049975], B103 [NC_004165], Nf [NC_049976], Goe6 [NC_049965], BSTP4 [MW354668.1], phi29 [NC_011048], PZA [NC_001423], BSTP6 [MW354670.1], GA1 [NC_002649], Gxv1 [NC_049974], Stitch [NC_031032], phi105 [NC_004167], Ray17 [MH752385.1], SPP1 [NC_004166], 049ML003 [MN176228.1], 019DV004 [MN176221.1], 049ML001 [MN176227.1], 000TH010 [MN176219.1], PM1 [NC_020883], 274BB002 [MZ501264.1], 276BB001 [MN176231.1], 056SW001B [MN176230.1], 019DV002 [MN176220.1], 268TH004 [MW394467.1], BSTP12 [MW354678.1], BSP7 [MH707430.1], Goe12 [MT601273.1], BUCT082 [MZ969646.1], Goe11 [MT601272.1], Goe13 [MT601274], phi3T [KY030782], SPO1 [NC_011421], SPbeta [NC_001884], SP8 [MW001214.1], SP10 [NC_019487], Goe2 [KY368639.1], CampHawk [NC_022761], SIOphi [NC_042133], phi18 [MZ751040.1], Goe10 [MT601271.1], Goe9 [MT601270.1], BSP38 [NC_048726], BSTP3 [MW354667], BSP10 [MF422185.1], 015DV002 [MW419086.1], 000TH009 [MW419085.1], 000TH008 [MW419084.1], phiNIT1 [NC_021856.1], BSP9 [MG000860.1], Goe3 [NC_048652], 015DV004 [MW419087.1], Grass [NC_022771], 278BB001 [MZ501265.1], Goe7 [MN043730.1], 043JT007 [MZ501263.1], 010DV005 [MZ501261.1], 010DV004 [MZ501260.1], 035JT004 [MZ501262.1], SP15 [NC_031245], AR9 [NC_031039], PBS1 [NC_043027], which are publically available in the GenBank [https://www.ncbi.nlm.nih.gov/genbank/].

## Abbreviations

BSPs: *B. subtilis* group species-infecting phages
NCBI: National Center for Biotechnology Information
BLAST: Basic Local Alignment Search Tool
ICTV: International Committee on Taxonomy of Viruses
MCP: Major Capsid Protein
PGA: Poly-Gamma-Glutamate
VIRIDIC: Virus Intergenomic Distance Calculator
