# Supplementary material for: Genomic diversity and comprehensive taxonomical classification of 61 Bacillus subtilis group member infecting bacteriophages, and the identification of ortholog taxonomic signature genes
Source: BMC Genomics. 2022 Dec 16;23:835. doi: 10.1186/s12864-022-09055-w (PMC9756591; doi:10.1186/s12864-022-09055-w)
Supplement: Supplementary file 1 — Additional file 1. Existing and newly proposed classifications of 61 BSPs. [file 12864_2022_9055_MOESM1_ESM.docx]

**Additional file 1**. Existing and newly proposed classifications of 61 BSPs.

| Family/Class | Existing and new subfamily | Existing and new genera | ICTV-accepted *B.* virus species | Name of the proposed species [*B.* phages] | No. of species | Total no. of phages |
| --- | --- | --- | --- | --- | --- | --- |
| *Myoviridae* | 0 | *Pemunavirus* | PM1 |  | 1 | 4 |
|  |  | *Takahashivirus* | PBS1(AR9) | | 1 |  |
|  |  | *Thornevirus* | SP15 |  | 1 |  |
| *Caudoviricetes* | ***New subfamily2*** | *Spbetavirus* | SPbeta |  | 1 | 18 |
|  |  | *New genus7* |  | BUCT082 | 1 |  |
|  |  | *New genus8* |  | Goe11, Goe12, Goe13 | 3 |  |
|  |  | *New genus9* |  | phi3T | 1 |  |
|  |  | *Spizizenvirus* | phi105 |  | 1 |  |
| *Siphoviridae* | ***New subfamily1*** | *New genus1* |  | Ray17 | 1 |  |
|  |  | *New genus2* |  | 000TH010, SPP1, 049ML001(049ML003) | 3 |  |
|  |  | *New genus3* |  | 268TH004(276BB001, 274BB002), 056SW001B, 019DV002(019DV004) | 3 |  |
| *Podoviridae* | 0 | *New genus4* |  | BSP7(BSTP12) | 1 | 2 |
| *Herelleviridae* | *Bastillevirinae* | *Grisebachstrassevirus* | vBBsuMGoe3(vB_BveM-Goe7) | | 1 | 26 |
|  |  | *Jeonjuvirus* | BSP38 |  | 1 |  |
|  |  | *Nitunavirus* | Grass  phiNIT1 | BSP9, BSP10(BSTP3), 035JT004, 278BB001, 043JT007 (010DV004, 010DV005) | 7 |  |
|  |  | *Siophivirus* | SIOphi |  | 1 |  |
|  |  | *New genus5* |  | 000TH008(000TH009), 015DV002, 015DV004 | 3 |  |
|  | *Spounavirinae* | *Okubovirus* | CampHawk, SPO1 | SP8, Goe9, Goe10, Goe2, phi18 | 7 |  |
|  |  | *New genus6* |  | SP10 | 1 |  |
| *Salasmaviridae* | *Northropvirinae* | *Claudivirus* | Stitch |  | 1 | 11 |
|  | *Picovirinae* | *Salasvirus* | Gxv1, PZA, phi29, Goe6 | BSTP4(BSTP6) | 4 |  |
|  |  | *Beecentumtrevirus* | Goe1, B103, Nf |  | 3 |  |
|  | *Tatarstanvirinae* | *Gaunavirus* | GA1 |  | 1 |  |
| Total | 7 | 23 | 21 | 28 | 49 | 61 |
