# Supplementary figures and images for: Genomic diversity and comprehensive taxonomical classification of 61 Bacillus subtilis group member infecting bacteriophages, and the identification of ortholog taxonomic signature genes
Source: BMC Genomics. 2022 Dec 16;23:835. doi: 10.1186/s12864-022-09055-w (PMC9756591; doi:10.1186/s12864-022-09055-w)

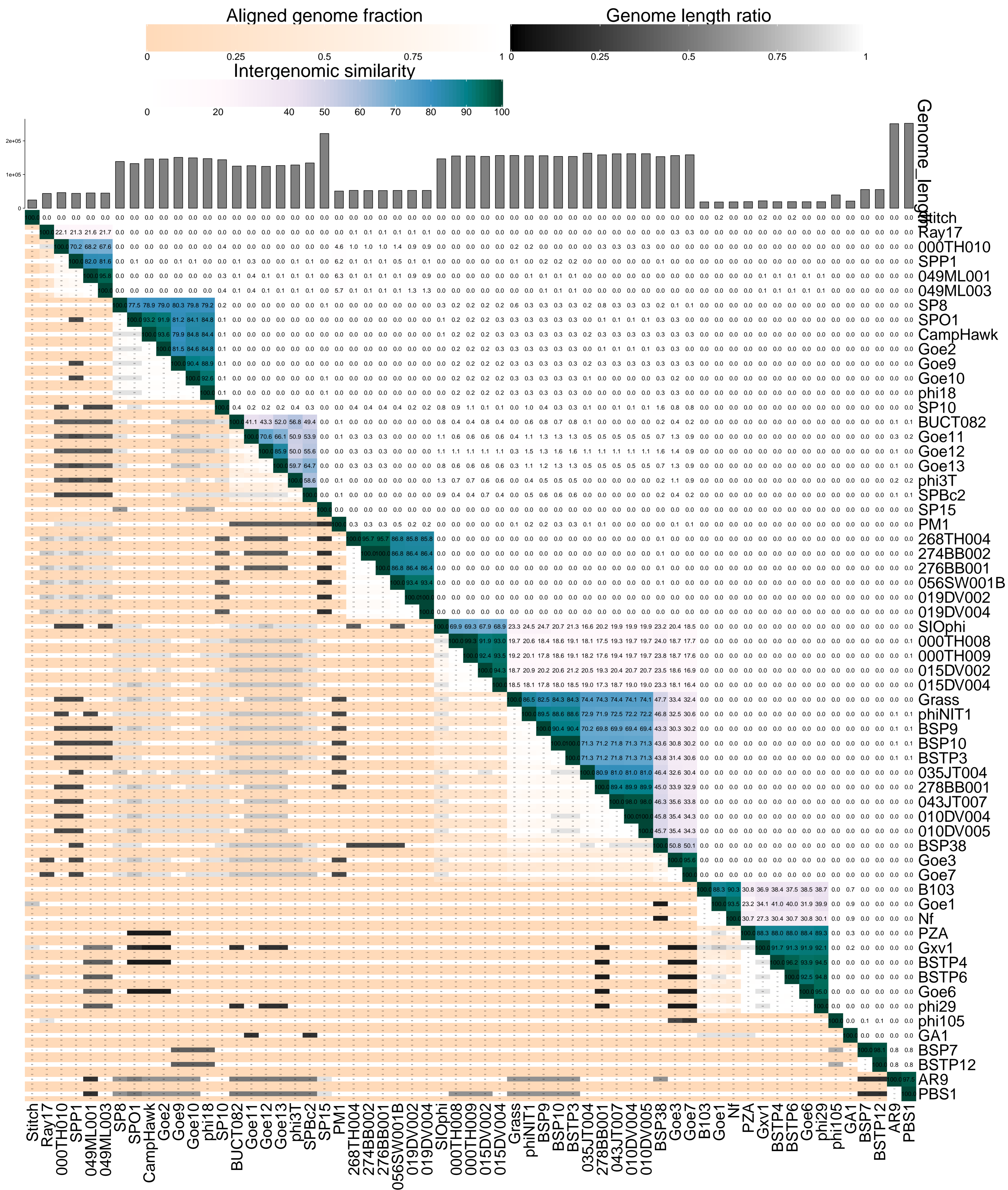

Supplement: Supplementary file 2 — Additional file 2. Intergenomic similarities of 61 BSPs. [file 12864_2022_9055_MOESM2_ESM.pdf]
